# Memory B Cells and CD8+ Lymphocytes Do Not Control Seasonal Influenza A Virus Replication after Homologous Re-Challenge of Rhesus Macaques

**DOI:** 10.1371/journal.pone.0021756

**Published:** 2011-06-29

**Authors:** Timothy D. Carroll, Shannon R. Matzinger, Linda Fritts, Michael B. McChesney, Christopher J. Miller

**Affiliations:** 1 Center for Comparative Medicine, University of California Davis, Davis, California, United States of America; 2 California National Primate Research Center, University of California Davis, Davis, California, United States of America; 3 Department of Pathology, Microbiology, and Immunology, School of Veterinary Medicine, University of California Davis, Davis, California, United States of America; 4 Department of Pathology and Laboratory Medicine, School of Medicine, University of California Davis, Davis, California, United States of America; Instituto Butantan, Brazil

## Abstract

This study sought to define the role of memory lymphocytes in the protection from homologous influenza A virus re-challenge in rhesus macaques. Depleting monoclonal antibodies (mAb) were administered to the animals prior to their second experimental inoculation with a human seasonal influenza A virus strain. Treatment with either anti-CD8α or anti-CD20 mAbs prior to re-challenge had minimal effect on influenza A virus replication. Thus, in non-human primates with pre-existing anti-influenza A antibodies, memory B cells and CD8α^+^ T cells do not contribute to the control of virus replication after re-challenge with a homologous strain of influenza A virus.

## Introduction

Seasonal influenza A virus infection is a highly contagious, acute respiratory tract disease of humans that causes substantial morbidity and mortality, particularly among the young, old, and immunocompromised [1]. After infection of people with an antigenically novel influenza A virus strain there is a 2–3 day period of virus replication and the full range of adaptive immune responses develops in response to the antigen produced [2], [3]. Influenza-specific antibodies are detected within 7 to 12 days of infection and gradually decline over the first 6 months post infection. Neutralizing antibodies specific for influenza hemagglutinin (HA) and neuraminidase (NA) correlate with protection from disease after exposure to a homologous influenza A virus [2]. Although most humans mount T cell responses to the immunodominant Matrix 1 protein after natural infection [4]; the human T cell response seldom extends to the other influenza A virus proteins [4]. Further, the role of antiviral T cell responses in controlling influenza A virus replication in people is undefined. Humans previously infected with one strain of influenza A virus are solidly protected from disease upon subsequent exposure to the homologous influenza A virus and this protection is associated with the presence of high titer antiviral antibodies [5]. Upon re-exposure to a homologous virus, virus replication is either completely blocked or severely blunted with no virus detectable after 48 hours. The nature of the immunity that provides this protection is not fully understood although there is little time for the expansion of memory T cells or the elaboration of humoral and cellular effector molecules by antigen-specific lymphocytes.

Immunity to human influenza viruses is often studied in mice and ferrets. Human influenza viruses normally replicate efficiently in mice only after adaptation [6] but ferrets are highly susceptible to infection with human influenza viruses and appear to better recapitulate human innate immunity, disease severity and transmissibility than mice [7], [8], [9]. Guinea pigs are also susceptible to human influenza infection and they have been used to study human influenza A virus transmission [10]. Nonhuman primate models are less often used in influenza research but they are commonly employed in AIDS research and are excellent models of the human immune and respiratory systems due to their relatively close phylogenetic relationship with people. Macaques are naturally and experimentally infected with human influenza A viruses with varying degrees of morbidity [11], [12], [13]. The kinetics of viral replication and the nature of the antiviral immune response in experimentally infected humans [3] and macaques [12] are similar, as strain-specific CD4^+^ and CD8^+^ T cell and antibody responses arise within 14 days of infection. Human seasonal influenza A viruses infect and replicate in the respiratory tract of macaques causing either asymptomatic or mild clinical infections [11], [12], [14]. The pandemic avian H5N1 [15] and 1918 H1N1 viruses [16] cause acute respiratory distress syndrome in macaques that is very similar to humans.

It has been shown that rhesus macaques previously infected with H3N2 Aichi influenza A virus are protected from homologous re-challenge 90 days later to the point that no infectious virus can be isolated. [11]. Thus influenza A virus infection of rhesus macaques induces potent antiviral immune effector mechanisms that can effectively block virus replication upon re-exposure. While it is generally accepted that influenza A virus hemagglutinin (HA) specific antibodies protect against rechallenge with antigenically matched viruses, the relative contribution of antibodies and other immune effector mechanisms to control of influenza virus replication in the respiratory tract is unknown. In the current study we administered either an anti-CD20 B cell depleting mAb or an anti-CD8α T cell and NK cell depleting mAb to rhesus macaques prior to their second experimental inoculation with a human seasonal influenza A virus strain. Despite the near complete depletion of peripheral CD20+ B cells or CD8+ T cells and the lack of an anamnestic antibody response in the B cell depleted animals, the level of viral replication in the intact and lymphocyte depleted animals were similar.

## Methods

### Ethics Statement/Animals

All animals used in this study were adult rhesus macaques (Macaca mulatta) housed at the California National Primate Research Center in accordance with the recommendations of the Association for Assessment and Accreditation of Laboratory Animal Care International Standards and with the recommendations in the Guide for the Care and Use of Laboratory Animals of the National Institutes of Health. The Institutional Animal Use and Care Committee of the University of California, Davis, approved these experiments (Protocol #11479). For blood collection, animals were anesthetized with 10 mg/kg ketamine hydrochloride (Park-Davis) injected i.m. For virus inoculation and respiratory secretion sample collection, animals were additionally anesthetized with 15–30 µg/kg Domitor (Orion Pharma) injected i.m., and anesthesia was reversed with 0.07–0.15 mg/kg Antisedan (Pfizer Animal Health) injected i.m. All efforts were made to minimize suffering. Details of animal welfare and steps taken to ameliorate suffering were in accordance with the recommendations of the Weatherall report, “The use of non-human primates in research”. Animals were housed in an air-conditioned facility with an ambient temperature of 21–25°C, a relative humidity of 40%–60% and a 12 h light/dark cycle. Animals were individually housed in suspended stainless steel wire-bottomed cages and provided with a commercial primate diet. Fresh fruit was provided once daily and water was freely available at all times.

### Monkey lymphocyte depletion, inoculation, sample processing and analysis of lymphocyte populations in blood

Nine animals assigned to 3 experimental groups were challenged with a previously described human influenza A virus isolate, A/Memphis/7/2001 (H1N1) [12], and re-challenged 4–8 months later using the same virus stock (Tables 1 and 2). Using methods previously described [17], [18], group A macaques (n = 3) were treated with a CD8α^+^ lymphocyte (CD8^+^ T cells and NK cells) depleting mAb (cM-T807, Centocor, Malvern, Pa.; 50 mg/kg; IV infusion) 3 days prior to re-challenge. Group B macaques (n = 5) were treated with a CD20^+^ B cell depleting mAb (rituximab, Genentech, Inc., South San Francisco, CA; 50 mg/kg; IV infusion) 28, 14, and 3 days prior to day of re-challenge.

**Table 1:** IgG titers in plasma after the first and second inoculations withA/Memphis/7/01.

			Weeks after Influenza A virus inoculation					
			1st inoculation		2nd inoculation			
Animal Number	Months between inoculations	Treatment	0	2	0^a^	1	2	4
30924	4	None	800^b^	80,000	160,000	640,000	640,000	640,000
30933	7	None	800	32,000	20,000	640,000	160,000	160,000
33470	8	None	800	64,000	40,000	320,000	320,000	160,000
MEAN			800	58,667	73,333	533,333^c^	373,333^c^	320,000
30811	7	α-CD8	800	64,000	40,000	320,000	640,000	640,000
30831	7	α-CD8	800	64,000	80,000	320,000	640,000	640,000
30851	7	α-CD8	800	160,000	80,000	320,000	640,000	640,000
MEAN			800	96,000	66,667	320,000^c^	640,000^c^	640,000
30616	7	α-CD20	800	64,000	160,000	160,000	160,000	160,000
30921	7	α-CD20	800	80,000	80,000	80,000	160,000	160,000
35125	4	α-CD20	800	32,000	40,000	40,000	80,000	160,000
MEAN			800	58,667	93,333	93,333^c^	133,333^c^	160,000

a = day of re-inoculation with A/Memphis/7/01.

b = endpoint dilution titers to whole disrupted A/Memphis/7/01. A titer of 1∶800 indicates the sample was below the cutoff established by screening plasma from A/Memphis/7/01 naïve animals.

c = day 7 PC titers of the groups are significantly different (p = 0.033, **Kruskal-Wallis test**) and the difference between the untreated and B cell depleted animal groups is also significant (p<0.05, Dunn's multiple comparison test.).

**Table 2:** HI titers in plasma after the first and second inoculations with A/Memphis/7/01.

			Weeks after Influenza A virus inoculation					
			1st inoculation		2nd inoculation			
Animal Number	Months between inoculations	Treatment	0	2	0^a^	1	2	4
30924	4	None	8^b^	128	320	1280	1280	1280
30933	7	None	8	32	64	1024	1024	2048
33470	8	None	8	64	256	512	1024	1024
MEAN			8	75	213	939	1109	1451
30811	7	α-CD8	8	128	80	640	2560	2560
30831	7	α-CD8	8	128	320	640	2560	2560
30851	7	α-CD8	8	1024	1280	1280	1280	2560
MEAN			8	427	560	853	2133	2560
30616	7	α-CD20	16	128	512	512	512	1024
30921	7	α-CD20	4	128	512	512	1024	2048
35125	4	α-CD20	4	16	128	128	512	512
MEAN			8	91	384	384	683	1195

a = day of re-inoculation with A/Memphis/7/01.

b = HA Inhibition titer to A/Memphis/7/01.

The human influenza A virus isolate used in this study, A/Memphis/7/2001 (H1N1), was generously provide by Richard Webby at the St. Jude's Children Hospital, Memphis, TN. This isolate was isolated on MDCK and was not passaged further prior to expansion in MDCK cells (American Type Culture Collection, Manassas, VA) to produce the virus stock used for animal inoculations. The virus stock had a titer of 10^6.5^ TCID_50_/ml on MDCK cells using the method of Reed and Muench [19]. The intranasal/intratracheal/conjunctival influenza A virus inoculation procedure and the respiratory secretion sample collection procedure have been previously described [12]. For both the initial and re-challenge inoculations, animals were inoculated with 6 ml of the virus stock instilled into the trachea, 1 ml of virus stock dripped intranasally, and a drop of virus stock in each conjunctiva. Blood samples were collected on days −28, −21, −14, 0, 7, 14 and 28 relative to the day of influenza A virus re-challenge. The percentage of CD3^+^ CD8^+^ T cells, CD3^−^ CD16^+^ NK cells and CD20^+^ B cells in peripheral blood was determined by flow cytometric analysis as previously described [17], [18].

### Titration of infectious influenza virus and viral RNA in respiratory secretions

The 50% tissue culture infectious dose (TCID_50_) of infectious virus in respiratory secretions was determined by end-point culture on MDCK cells as previously described [12]. Further, the amount of virion-associated RNA (vRNA) in respiratory secretions was determined by RT-PCR as previously described [12].

### Influenza Antibody ELISA and HI assays

Titers of anti-influenza antibodies were determined by a modification of a method previously described [12], [20].

### Tracheal Sample Cytokine mRNA Expression Levels

The method for assessment of host gene expression in tracheal secretions has been published [12]. Briefly, total RNA was isolated from the cellular pellets with TRIzol® (Invitrogen) according to manufacturer's instructions. RNA samples were DNase-treated and cDNA was prepared using random hexamer primers (Amersham-Pharmcia Biotech Inc.) and SuperScript III reverse transcriptase (Invitrogen). Cytokine mRNA levels were determined by RT-PCR as described previously [21], [22]. The GAPDH housekeeping gene and the target gene from each sample were run in parallel in the same plate. The reaction was carried out in a 96-well optical plate (Applied Biosystems) in a 25 µl reaction volume containing 5 µl cDNA plus 20 µl Mastermix (Applied Biosystems). All sequences were amplified using the 7900 default amplification program. The results were analyzed with the SDS 7900 system software, version 2.1 (Applied Biosystems). Cytokine mRNA expression levels were calculated from normalized ΔC_T_ values. C_T_ values correspond to the cycle number at which the fluorescence due to enrichment of the PCR product reaches significant levels above the background fluorescence (threshold). In this analysis, the C_T_ value for the housekeeping gene (GAPDH) is subtracted from the C_T_ value of the target (cytokine) gene (ΔC_T_). In general, the ΔC_T_ value for the influenza A-infected sample is then subtracted from the pre-infection ΔC_T_ value (ΔΔC_T_). Assuming that the target gene (cytokine) and the reference gene (GAPDH) are amplified with the same efficiency (data not shown), the increase in cytokine mRNA levels in test samples is then calculated as follows: increase = 2*^−ΔΔC^_T_* (user bulletin no. 2, ABI Prism 7700 Sequence Detection System: Applied Biosystems). Cytokine mRNA levels are expressed as the increase or decrease relative to the level for that cytokine in the individual monkey's pretreatment secretion sample. Because the mRNA expression level of housekeeping genes such as GAPDH can change under activating conditions, we were careful to use the same input amount of RNA for experimental samples in the PCR reactions. Regardless of the sample was collected the same input amount of RNA consistently resulted in similar PCR amplification (C_T_) values for GAPDH. Therefore, GAPDH expression in trachea was not differentially regulated among the animals in this study.

### Statistical Analysis

Statistics are reported as the mean and the standard error of the mean for each group using Prism 5.0a software (GraphPad) and data are presented as the probability and test used for analysis. Mean levels of lymphocyte subsets, vRNA and TCID_50_ in the treated groups were independently compared to the untreated control animal group with a one-tailed unpaired T test. Linear regression analysis and Pearson's Correlation analysis were used to define the relationship between serum antibody titers and virus replication and the results of both analyses are reported.

## Results

### Antibody responses and Influenza A virus replication after the initial inoculation

**Figure 1:**
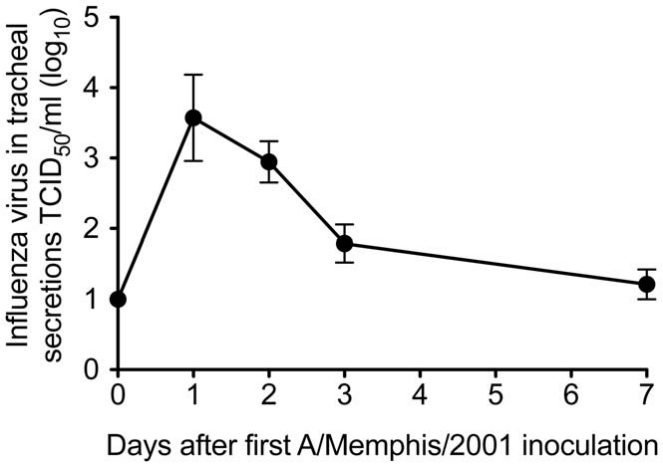
Influenza virus replication in the lower respiratory tract after A/Memphis/7/01 inoculation. Mean infectious virus titer in tracheal secretions (TCID_50_/ml) of nine inoculated rhesus macaques.

Prior to inoculation, 8 of 9 animals had plasma IgG antibody titers to A/Memphis/7/01 that were below the cutoff (1∶800) for the assay (Table 1) and HI titers ranged from 1∶4 to 1∶16 (Table 2). In contrast to the other 8 monkeys, both pre-inoculation plasma samples from monkey 35125 had O.D. values at 1∶800 that were just at the cut-off, consistent with the presence of low-titer A/Memphis/7/01-specific binding antibodies on the day of challenge (Table 1), however A/Memphis/7/01-specific HI antibodies of this animal were undetectable at the lowest dilution (1∶4) tested (Table 2). By 2 weeks after inoculation, all 9 animals had made strong anti-influenza antibody responses, binding IgG antibody titers ranged from 1∶32,000–1∶160,000 (Table 1) and HI titers ranged from 1∶16–1∶1024 (Table 2). After the first experimental inoculation with A/Memphis/7/01, infectious virus was isolated from the tracheal secretions of all 9 animals in this study. As we have previously described, virus replication peaked 24–48 hours after inoculation (mean peak titer of 10^4.1^ TCID_50_/ml; Fig. 1), steadily declined but was detectable in all 9 animals at day 3 PI, until day 7 when infectious virus could not be isolated from any of the animals (Fig. 1). Assays to quantify influenza virus RNA in the secretions of these 9 animals were not performed after the initial infection, however we previously reported [12] that after an initial challenge with A/Memphis/7/01, the peak vRNA levels in tracheal secretions of 3 animals ranged from 5.4–6.4 log10 vRNA copies/ml secretions (Table 3).

**Table 3:** Influenza virus RNA and host gene mRNA levels in tracheal secretions 24 hours after initial A/Memphis/7/2001 (H1N1) challenge; H1N1 re-challenge; or challenge with heat-inactivated H1N1.

		mRNA expression fold change relative to pre exposure			
Animal Number	Peak log_10_ vRNA copies/ml	IFN-α	OAS	MxA	IL-6
*No Treatment prior to H1N1 re-challenge*					
30924	3.7	−5.1	4.3	3.2	10.9
30933	3.9	NA^a^	27.6	11.0	3.4
33470	4.6	162.3	32.3	15.5	13.6
Mean	4.1	78.6	21.4	9.9	9.3
*Anti-CD8 Treatment prior to H1N1 re-challenge*					
30811	4.8	NA	NA	NA	86.9
30831	3.1	3.4	5.1	3.5	22.7
30851	2.2	2.5	3.9	5.0	12.6
Mean	3.4	2.9	4.5	4.3	40.7
*Anti-CD20 treatment prior to H1N1 re-challenge*					
30616	2.5	−2.7	2.5	1.5	22.1
30921	4.2	NA	NA	NA	NA
35125	4.3	−34.6	5.7	16.4	6.0
Mean	3.7	−18.6	4.1	8.9	14.0
*Initial H1N1 challenge* b					
33073	5.8	3910.6	53.7	29.4	287.8
33178	5.4	11340.0	101.8	15.3	72.5
34421	6.4	1606.1	65.4	19.2	5786.6
Mean	5.9	5618.9	73.6	21.3	2048.9
*Initial challenge with Heat-inactivated H1N1* b					
31392	2.9	−11.3	1.7	5.3	−2.8
31625	3.5	40.6	2.0	5.5	NA
31705	2.4	1.1	1.8	2.6	−4.3
31864	3.1	−14.0	−1.9	10.8	−2.5
Mean	3.0	4.1	0.9	6.0	−3.2

a = target gene mRNA levels could not be determined due to poor amplification of GAPDH mRNA.

b = data adapted from *Carroll et al, J. Immunol. 2008* [12].

### Anti-CD8α and anti-CD20 mAbs effectively deplete targeted lymphocyte populations in the blood of treated animals

**Figure 2:**
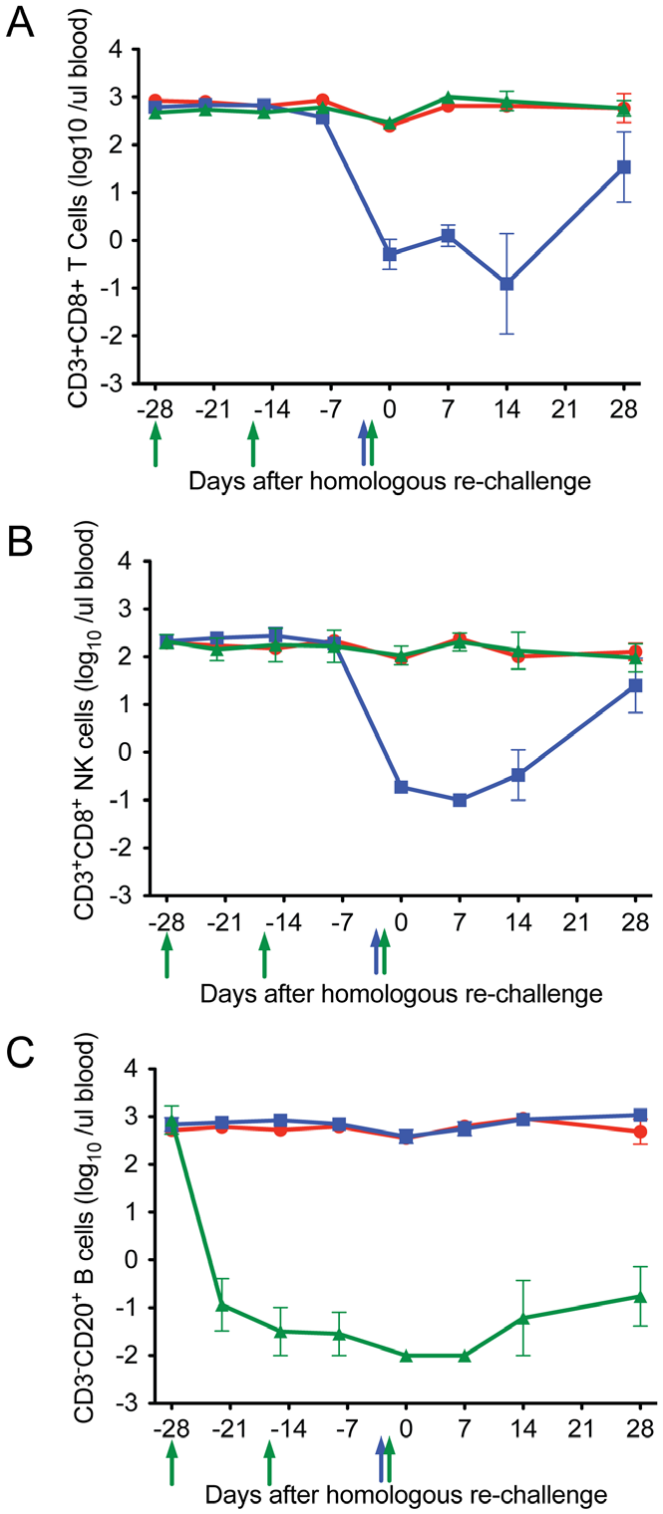
The effect of anti-CD20 and anti-CD8α infusion on CD20^+^ B cells, CD3^+^ CD8a^+^ T cells and CD3^−^ CD8a^+^ NK cells in blood of animals at the time of influenza virus re-challenge. a) CD20^+^ B cells in blood, b) CD3+ CD8a^+^ T cells and, c) CD3^−^ CD8a^+^ NK cells. Green symbols and lines denote the anti-CD20 monkeys (n = 3), blue symbols and lines denote the anti-CD8α-treated monkeys (n = 3) and red symbols and lines denote un-treated monkeys (n = 3). Day 0 indicates the day of influenza re-challenge. Anti-CD20 was infused 28, 14, and 3 days prior to the day of influenza re-challenge, anti-CD8 was infused 3 days prior to virus inoculation. Arrows indicate the timing of the antibody infusions; blue arrow indicates anti-CD8a infusion and green arrows indicate anti-CD20 infusions.

Adult rhesus macaques previously infected with A/Memphis/7/01 were infused with either anti-CD8α (n = 3), anti-CD20 mAbs (n = 3) or left untreated (n = 3) prior to re-challenge with A/Memphis/7/01 (Tables 1 and 2, Fig. 2). On the day of influenza A virus re-challenge, 28 days after the first anti-CD20 infusion (Fig. 2), the mean number of circulating CD20^+^ B cells in the 3 treated animals (0.04±0.2 B cells/µl blood) was 1000 fold lower compared to untreated animals (393±70 B cells/µl blood) and this difference was highly significant (p<0.0001, unpaired one tail T test). Three days after anti-CD8 infusion (Fig. 2), on the day of re-challenge, the mean numbers of CD8^+^ T cells and CD8^+^ NK cells in the blood of the 3 treated animals (0.8±0.4 CD8^+^ T cells/µl blood, 0.2±0.5 NK cells/µl blood) were also reduced approximately 1000 fold compared to the 3 untreated animals (268±131 CD8^+^ T cells/µl blood, 111±23 CD8^+^ NK cells/µl blood) and these differences were significant (p<0.0001 for CD8+ T cells and p<0.0001 for NK cells, unpaired one tail T tests). The CD20+ B cell and CD8a+ lymphocyte populations remained depleted from the day of influenza re-challenge to 7 days PC (Fig. 2), completely blocking anamnestic responses by these lymphocyte subsets for the first week after re-challenge (see below). Further we, and others, have shown that if the anti-CD20 and anti-CD8 Mabs effectively deplete peripheral lymphocyte subsets then more than 90% of their target cells are also depleted from the tissues of rhesus macaques [18], [23], [24], [25], [26], [27], [28]. Thus, although the level of lymphocyte depletion in the respiratory tract was not examined in the above studies, it is reasonable to assume that the Mabs depleted most of the target lymphocytes from tissues of the animals in the present study.

### Influenza A virus replication is well-controlled upon homologous re-challenge of untreated animals

**Figure 3:**
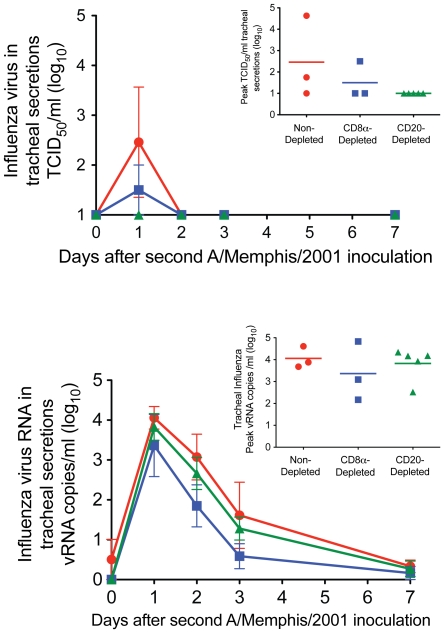
Influenza virus replication in the lower respiratory tract after A/Memphis/7/01 inoculation. A) Mean infectious virus titer in tracheal secretions (TCID_50_/ml). Inset; mean tracheal secretion peak TCID_50_ (B) Mean vRNA copy number in tracheal secretions (Log_10_ copies/ml). Inset; mean tracheal secretion peak vRNA. • No lymphocyte depletion before re-challenge (n = 3) ▪ CD8α^+^ T cell and NK cell depletion before re-challenge (n = 3) ▴ CD20^+^ B cell depletion before re-challenge (n = 3).

On the day of re-challenge with A/Memphis/7/01, the 3 untreated animals had a mean A/Memphis/7/01 HI titer of 1∶213 (range 1∶64–1∶320) (Table 2). After homologous re-challenge, low levels of virus could be isolated from the tracheal secretions of only 2 of the 3 animals (Fig. 3a) compared to the mean peak titer of 10^4.1^ TCID_50_/ml (Fig. 1) in the naïve animals after the first A/Memphis/7/01 inoculation. On days 1 and 2 after homologous re-challenge, vRNA was detected in tracheal secretions of all 3 untreated animals at very low levels (mean peak titer of 10^4.1^ vRNA copies/ml). Based on our previously published [12] and shown in Table 3, we expect influenza RNA levels of 10^5^–10^6^ copies/ml of tracheal secretions 24–48 hours after A/Memphis/7/01 inoculation of naïve animals. We have also shown that 24 hours after inoculation of 4 naïve animals with heat-killed A/Memphis/7/01, vRNA levels ranged from 10^2.4^–10^3.5^ vRNA copies/ml secretions [12] (Table 3). Because influenza A virus vRNA levels in the secretions after homologous re-challenge are higher (0.6–1.7 log10) than after inoculation with heat-killed virus, some low level replication is occurring after re-challenge. Thus, based on both infectious virus levels and vRNA levels in tracheal secretions it is clear that influenza A virus replication was very well-controlled in rhesus macaques that were re-challenged 4 months after being infected with the same virus.

### Limited role of memory CD8^+^ or CD20^+^ lymphocytes in control of viral replication after homologous influenza A virus re-challenge

On the day of re-challenge, the 3 anti-CD20 mAb-treated animals had a mean A/Memphis/7/01 HI titer of 1∶384 (range 1∶280–1∶512); and the 3 anti-CD8α mAb -treated animals had a mean A/Memphis/7/01 HI titer of 1∶560 (range 1∶80–1∶1280) (Table 2). After A/Memphis/7/2001 re-challenge, the mean peak virus titer (TCID_50_/ml) and the mean area under the curve (AUC; virus titer from day 0 to day 7 after re-challenge) in the tracheal secretions of the anti-CD8α and anti-CD20 mAb treated animals were separately compared to the untreated animals.

Similar to the control group, animals treated with either anti-CD8α or anti-CD20 mAbs prior to re-challenge had very little detectable influenza A virus replication (Fig. 3). In fact, infectious virus was isolated from only 1 CD8α-depleted animal; and both the peak TCID_50_/ml (Fig. 3a, inset) and the AUC level in this animal were very low (Fig. 3a). Further on days 1 and 2 after re-challenge, similar levels of vRNA (log_10_ copies/ml) were detected in tracheal secretions of the anti-CD8α-, anti-CD20 mAb-treated and untreated animals (Fig. 3b).

**Figure 4:**
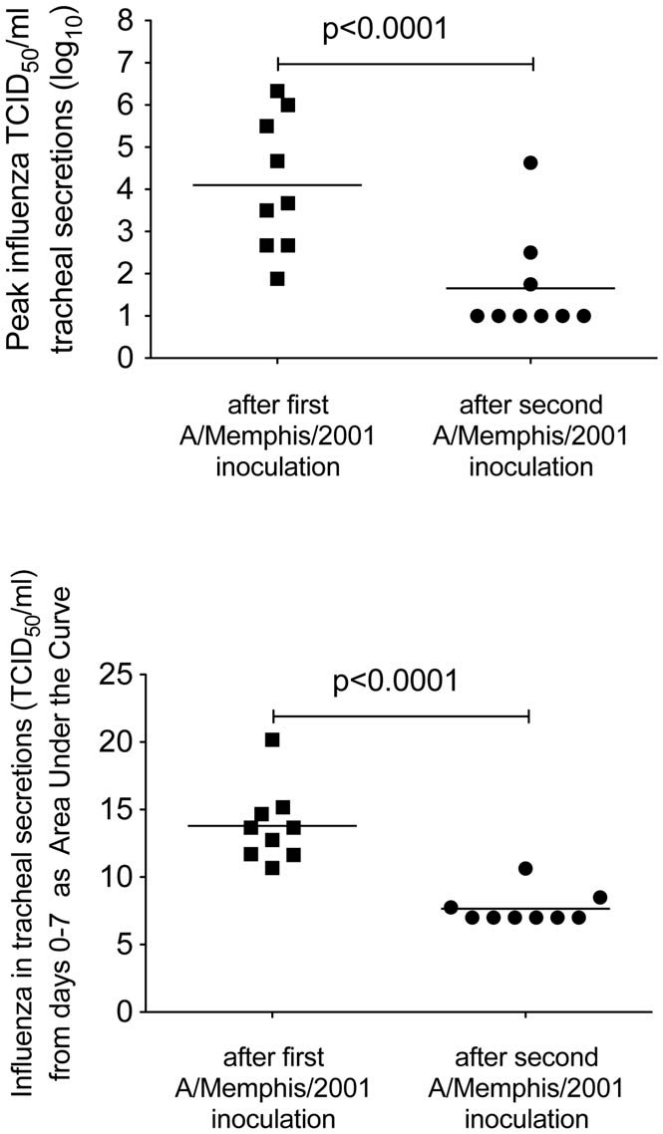
Comparison of influenza virus replication in the lower respiratory tract after the first and second A/Memphis/7/01 inoculations. (A) Mean peak infectious virus titer in tracheal secretions (TCID_50_/ml). (B) Mean AUC of the infectious virus titer in tracheal secretions from day 0 to day 7 after re-challenge.

To gauge the ability of the different animal groups to control virus replication after homologous re-challenge relative to the first A/Memphis/7/01 inoculation, peak infectious titers and AUC of the infectious virus titer from day 0 to 7 were compared (Fig. 4). As the levels of virus replication were similar in the treated and untreated animals after rechallenge, all animals were grouped for this comparison. Both the peak TCID_50_ and the TCID_50_ AUC were significantly lower after re-challenge than after the initial infection, clearly illustrating the effectiveness of anti-influenza immunity against matched virus strains.

### Limited role of local innate immune responses in control of viral replication after homologous influenza A virus re-challenge

The mRNA levels of IFN-α, MxA, OAS and IL-6 in tracheal secretions of an animal at 24 hours after re-challenge were compared to levels in the same animal 72 hours prior to re-challenge (Table 3). For comparison, the previously published data [12] for a similar experiment performed on 3 animals after their initial infection with A/Memphis/7/01 and on 4 animals after they were inoculated with heat inactivated A/Memphis/7/01 are shown in Table 3. In contrast to the high mRNA levels of all 4 genes in tracheal secretions of 3 animals after an initial H1N1 infection, target gene mRNA levels among the 9 study animals remained uniformly low (Table 3). In fact, after H1N1 re-challenge, host gene expression levels in the tracheal secretions of the all animals were similar, regardless of intervention (Table 3). These results indicate that local innate immune responses did not contribute to control of viral replication after homologous influenza A virus re-challenge even in the lymphocyte depleted animals.

### Pre-existing strain-specific anti-influenza antibody titers are unaffected by treatment with anti-CD8α or anti-CD20 mAbs

Prior to the initial challenge with A/Memphis/7/01 IgG antibodies were undetectable above the 1∶800 plasma dilution cut-off with the exception of 35125 (Table 1). In addition, none of the animals had plasma HI titers to A/Memphis/7/01 greater than 1∶16 before initial infection (Table 2). By day 14 after the first infection, anti-influenza IgG and HI antibody titers among the 9 study animals had increased dramatically. On the day of rechallenge, the anti-influenza antibody responses remained strong in all animals (Tables 1 and 2), despite the fact that the 3 anti-CD20 mAb treated animals were completely depleted of circulating CD20+ B cells prior to re-challenge. Thus, on the day of homologous influenza virus A re-challenge, the animals treated with anti-CD20, anti-CD8α, and the untreated animals had similarly high IgG and HI responses (Tables 1 and 2). The persistent strong antibody responses despite anti-CD20 treatment presumably reflect the 21-day half-life of plasma IgG and the fact that plasma cells were unaffected by the treatment due to the lack of CD20 expression [29], [30].

To understand the effect of the anti-CD20 mAb treatment on the anamnestic anti-influenza antibody responses after re-challenge IgG and HI antibodies were assessed. From the day of re-challenge to 7 days after re-challenge, there was no change in anti-influenza IgG and HI titers of the anti-CD20-mAb treated animals. In contrast anti-CD8α-mAb treated animals had a 4.8 fold increase in IgG antibody titers and a 1.5 fold increase in HI antibody titers, while the untreated animals had 4.4 fold increase in HI antibody titers and 7.3 fold increase in IgG antibody titers (Tables 1 and 2). Thus the CD20 lymphocyte depletion completely blocked the normal anamnestic antibody responses to homologous influenza A virus re-challenge.

### Relationship between HI titers on the day of homologous influenza A virus re-challenge and control of virus replication

**Figure 5:**
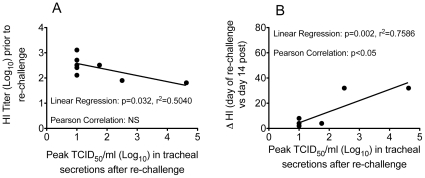
Correlation between serum HI titers and influenza virus replication in the lower respiratory tract after A/Memphis/7/01 re-challenge. A) HI titer on day of re-challenge and peak infectious virus titer in tracheal secretions after re-challenge. B) Fold change in HI titer from the day of re-challenge to 14 days later and peak infectious virus titer in tracheal secretions after re-challenge.

To better understand the relationship between HI titers on the day of A/Memphis/7/01 re-challenge and viral replication, linear regression and Pearson's correlation analyses were undertaken (Fig. 5a). Although infectious virus was undetectable in 6 of the 9 animals after homologous re-challenge, the 2 animals with the highest peak TCID_50_ had the lowest HI titers on the day of re-challenge. Thus, the linear regression analysis suggested there was significant negative correlation (p = 0.032 and r^2^ = 0.5) between the HI titer on the day of re-challenge and the level of viral replication after the re-challenge; however, Pearson's correlation analysis did not demonstrate a significant relationship. In addition, among the 6 animals with an intact B cell population on the day of challenge, there was significant positive correlation between the change in HI titer between the day of re-challenge and day 14 after re-challenge (p = 0.002 and r^2^ = 0.76, Pearson Correlation coefficient p<0.05) and peak TCID_50_ after re-challenge. Thus in the 2 animals with the highest detectable peak TCID_50_ there was a 32-fold change in HI titer between the day of re-challenge and day 14 after re-challenge. While in the 4 animals with undetectable infectious virus or very low peak TCID_50_ there was a ≤8-fold change in HI titer between the day of re-challenge and day 14 after re-challenge. Thus large increases in HI titer only occurred in animals that had demonstrable productive infection after re-challenge.

## Discussion

The results presented here demonstrate that, as in humans, rhesus monkeys control seasonal influenza A virus replication after secondary exposure to homologous virus. Further, IFN-α induced antiviral mediators, effector CD8^+^ T cells and anamnestic B cell responses do not seem to contribute to control of virus replication after homologous influenza A virus re-challenge of rhesus macaques (Fig. 3, Table 3). However, the level of strain-specific HI antibodies and anti-influenza IgG in plasma on the day of homologous influenza A virus re-challenge inversely correlated with the level of virus replication in the rhesus macaques (Fig. 5A). As the level of virus replication in lymphocyte-depleted animals was similar to intact animals regardless of which effector lymphocyte arm was depleted, it seems unlikely that the intact subset contributed to control of virus replication upon re-exposure. However, to confirm this conclusion an experiment that depletes both B cells and CD8a+ lymphocytes prior to infection could be conducted. These results strongly imply that anti-influenza antibodies in plasma at the time of homologous influenza A virus re-challenge are an immune correlate for the control of virus replication in these previously infected animals.

Epidemiologic studies in humans have correlated the incidence of influenza A virus infection with the level of pre-existing HI serum antibodies [31], [32], [33], [34] or binding anti-influenza antibodies [5] making it possible to estimate the titer of HI antibodies that is necessary to prevent virus replication and limit disease. In three successive epidemics of antigenically-drifted H3N2 influenza A viruses, the 50% protective HI titer (PT50) was estimated to be between 1∶12–1∶24, and no infections were recorded in persons with HI titers ≥1∶48 [33]. In addition, during an outbreak of infection with wild-type influenza A/Victoria/3/75 virus, 0 of 19 people with pre-epidemic HI titers >1∶40 became infected [35]. Perhaps most convincingly, a similar correlation was found in challenge experiments of immunized humans using a variety of influenza A virus strains, including the A/Hong Kong/68, with a calculated PT50 from 1∶18 to 1∶36 based on virus isolation or a 4-fold rise in plasma HI titer to the challenge virus as measures of infection [36]. In the rhesus macaques studied here, all animals had HI titers >1∶64 on the day of homologous influenza A virus re-challenge and they all controlled viral replication relative to their initial infection with A/Memphis/7/01. Of note, although the HI titer did correlate with the extent of viral replication and shedding, a serum HI titer of 1∶64 was not completely protective as 3 animals with HI titers >1∶64 had evidence of low level viral shedding (Fig. 3). As serum HI titer is the major immune correlate for control of influenza virus replication upon a second experimental exposure to homologous virus in rhesus macaques and humans [36], the immune effector mechanism responsible for protection from uncontrolled virus replication and disease may be similar in humans and macaques. It is important to recognize that the correlation between serum HI levels and protection does not imply causality, and neutralizing antibody must be present within the respiratory tract to prevent virus replication in the respiratory epithelium. In fact, we recently showed that in rhesus macaques immunized with an inactivated influenza vaccine (Fluzone) IgG anti-influenza antibody titers in the tracheal secretions and plasma of the animals 3 days prior to H1N1 challenge inversely correlated with virus replication after challenge [37]. Understanding the relative importance of local and systemic B-cell responses in maintaining a persistent level of protective HI antibody within the respiratory tract would facilitate the development of more effective influenza vaccines.

The 4–7 month interval between the first H1N1 influenza A virus infection and the homologous re-challenge of the rhesus monkeys in this study approximates the length of an influenza season, and thus the results reported here provide a ready explanation for resistance to a second infection with a homologous influenza A virus in a single season. However, this study did not attempt to identify the immune mechanisms responsible for long-term protection from homologous influenza A virus re-challenge. Some studies in humans suggest that HI titers remain above a PT50 of 1∶16–32 for at least 25–30 years. In fact people infected with the H1N1 subtype before 1950 were subsequently protected when this subtype reappeared in 1977, and similarly, people born before 1892 and infected with the H3N2 subtype were protected from disease when this subtype reappeared in 1968 [38], [39]. Persistent HI titers to H3 found in the serum of the individuals born before 1892 and persistent HI titers to H1 in persons born before 1950 are thought to account for this extraordinarily long-lived protection [38]. The mechanism behind this long-lived plasma antibody response is unknown but it is thought to be dependent on memory CD4+ T cells and memory B cells (reviewed in [40]). The persistence of these long-lived memory lymphocytes may be aided by repeated exposure to, and infections with, unrelated influenza A viruses; a hypothesis that could be directly tested in rhesus macaques that live for 20–30 years in captivity.

This study clearly shows that in primates, memory B cells and CD8α^+^ T cells do not contribute to the control of virus replication after re-challenge with a homologous H1N1 strain of influenza A. In future studies, administration of anti-CD8α, anti-CD4 or anti-CD20 depleting antibodies in rhesus macaques challenged with antigenically-drifted and heterosubtypic influenza A viruses could be used to define the nature of protective, vaccine-induced, and infection-induced immune responses to progressively divergent viruses. This information could then be used to rationally guide the development of universal influenza vaccines.
